# Prediction of microbe–disease association from the integration of neighbor and graph with collaborative recommendation model

**DOI:** 10.1186/s12967-017-1304-7

**Published:** 2017-10-16

**Authors:** Yu-An Huang, Zhu-Hong You, Xing Chen, Zhi-An Huang, Shanwen Zhang, Gui-Ying Yan

**Affiliations:** 1 Department of Information Engineering, Xijing University, Xi’an, 710123 China; 2 School of Information and Control Engineering, China University of Mining and Technology, Xuzhou, China; 3 College of Computer Science and Software Engineering, Shenzhen University, Shenzhen, 518060 China; 4 Academy of Mathematics and Systems Science, Chinese Academy of Sciences, Beijing, 100190 China

## Abstract

**Background:**

Accumulating clinical researches have shown that specific microbes with abnormal levels are closely associated with the development of various human diseases. Knowledge of microbe–disease associations can provide valuable insights for complex disease mechanism understanding as well as the prevention, diagnosis and treatment of various diseases. However, little effort has been made to predict microbial candidates for human complex diseases on a large scale.

**Methods:**

In this work, we developed a new computational model for predicting microbe–disease associations by combining two single recommendation methods. Based on the assumption that functionally similar microbes tend to get involved in the mechanism of similar disease, we adopted neighbor-based collaborative filtering and a graph-based scoring method to compute association possibility of microbe–disease pairs. The promising prediction performance could be attributed to the use of hybrid approach based on two single recommendation methods as well as the introduction of Gaussian kernel-based similarity and symptom-based disease similarity.

**Results:**

To evaluate the performance of the proposed model, we implemented leave-one-out and fivefold cross validations on the HMDAD database, which is recently built as the first database collecting experimentally-confirmed microbe–disease associations. As a result, NGRHMDA achieved reliable results with AUCs of 0.9023 ± 0.0031 and 0.9111 in the validation frameworks of fivefold CV and LOOCV. In addition, 78.2% microbe samples and 66.7% disease samples are found to be consistent with the basic assumption of our work that microbes tend to get involved in the similar disease clusters, and vice versa.

**Conclusions:**

Compared with other methods, the prediction results yielded by NGRHMDA demonstrate its effective prediction performance for microbe–disease associations. It is anticipated that NGRHMDA can be used as a useful tool to search the most potential microbial candidates for various diseases, and therefore boosts the medical knowledge and drug development. The codes and dataset of our work can be downloaded from https://github.com/yahuang1991/NGRHMDA.

**Electronic supplementary material:**

The online version of this article (doi:10.1186/s12967-017-1304-7) contains supplementary material, which is available to authorized users.

## Background

Mammalian hosts have a close relationship with microorganisms which colonize niches including the urogenital tract, skin, upper and lower respiratory tract, intestine and internal organs [1]. Many important biological interactions and processes arise from a diverse variety of microbes, and therefore human microbiome is emerging as an essential “organ” governing health and disease [2–4]. For example, commensal bacteria from around 500–1000 species inhabiting the skin have been reported to be involved in educating immune system in response to infection and injury, and maintaining homeostatic control of skin inflammation [5]. The presence of nearly 10^14^ bacterial cells from more than 10,000 microbial species in human internal environment provides diverse gene products which induce different biochemical and metabolic activities [6–8]. Even though the massive contribution of microbes has been revealed, a detailed understanding of mechanisms underlying host–microbe interactions and their impact on different human diseases remains largely elusive [9].

The composition of endogenous microbial community can undergo constant changes and differ from person to person owing to different environmental variable such as host diet [10, 11], season [12], smoking [13], hygiene and use of antibiotics [14]. The deviant compositions of microbial community can lead to varying degrees of damage to the tissues of hosts and further induces diverse diseases [15]. And the abundance distribution of microbes has also been reported to be associated with several human diseases [16]. For example, low microbial diversity can cause obesity and inflammatory bowel disease [17, 18], while high microbial diversity in the vagina is linked to bacterial vaginosis [19]. Pathogenic microbes can endure selective pressures of their environment with different strategies, and this genetically distinct population of microbes is usually regarded as contributor for different diseases such as allergic asthma [20], colorectal carcinoma [21], necrotizing enterocolitis [22, 23], atopic dermatitis [24] and psoriasis [25]. For example, Skov et al. have reported that the toxins from *Streptococcus* and *Staphylococcus aureus* can function as superantigens which boost the development of guttate psoriasis by bypassing the normal control of T cell activation [26]. Socransky et al. have observed that subgingival plaque is associated with several major microbial complexes including *Fusobacterium*, *Porphyromonas gingivalis*, *Prevotella* and *Treponema* [27]. Sze et al. have also identified an increase of the *Firmicutes phylum* and *Burkholderia* in patients with very sever chronic obstructive pulmonary disease (COPD) by Pyrotag sequencing [28].

With the development of experimental tools such as PCR, high-throughput sequencing and MALDI-TOF mass spectrometry (MS) as well as new sampling and culture strategies, much progress has been made towards discovering the mechanisms of microbial pathogenesis and microbe–disease associations [16, 29, 30]. Although an increasing amount has been discovered and recorded about the associations between microbes and diseases, technological hurdles remain to detect microbe–disease associations on a large scale [9]. Rather than a ‘one-bacterium, one-disease’ model, diseases are usually cased and influenced by the dynamic interplay between host and microbe and the complex activity of microbial community. Experiment-based methods for identifying microbe–disease associations usually need a long and densely sampled time series to observe many individuals with different traits because of different host pressures and the dynamic microbial behavior. In addition, the host–microbe interactions involved in different diseases are still hard to be verified as accidental or obligatory based on the transcriptomics [31].

Even though the regulatory mechanism by way of which microbial participators get involved is still not well known, further ventures into identification of microbe–disease associations would boost diagnostic and therapeutic support for the clinical management of patients. Knowledge about microbe–disease associations can provide valuable insights into understanding complex disease mechanisms. For example, gastric and duodenal ulcers and Whipple’s disease, which were considered as noninfectious in origin, have been reclassified as infectious ones after the identification of associated pathogenic organisms [32]. In addition, knowing the disease-causing microbes can also illuminate newer ways to promote disease diagnosis and therapy. For example, fecal microbiota transplantation has recently proved to be a safe and feasible treatment option for clostridium difficile infection (CDI) [14], which tries to rebuild healthy microbial community by reintroducing normal flora via donor feces. Detecting novel microbial participators engaging the disease development is clearly important for the application of this treatment. Predicting new microbe–disease associations is expected to select the most potential candidates for validation experiments and therefore to accelerate the researches and reduce cost. However, little effort has been made to develop prediction models for referring novel microbe–disease associations. Recently, the first database storing microbe–disease association data called HMDAD has been built by Ma et al. by manually curating from large-scale pubic literatures and the researchers discovered that the microbe-based disease network has strong overlaps with those disease network constructed based on genes, symptoms, chemical fragments and drugs. Specifically, HMDAD mainly focuses on non-infective diseases which are rarely clinically studied from a microbial perspective.

In this work, we have proposed a neighbor- and graph-based combined recommendation model for human microbe–disease association prediction (NGRHMDA). This model is mainly based on the assumption that functionally similar microbes tend to intertwine in the development of similar disease, similar with the basic hypothesis of recommended systems that users who owns the same/similar likings will like similar kinds of items. NGRHMDA model is combined by two separate recommendation model, one of which is neighbor-based collaborative filtering and the other is based on topological information of known microbe–disease bipartite graph. And this model combines symptom-based similarity and Gaussian kernel-based similarity for measuring disease and microbe similarity. To evaluate the effectiveness of the proposed model, two evaluation frameworks (i.e. lease-one-out and fivefold cross validations) have been implemented on HMDAD database, and the corresponding ROC curves have been computed. As a result, the ensemble model of NGRHMDA yielded an average AUC of 0.9023 ± 0.0031 for fivefold cross validation and AUC of 0.9111 for LOOCV, which increased at least 0.0169 and 0.0130 from the single models. In addition, the stability of the model was showed to be improved by combining. The prediction results showed additional disease similarity, like symptom-based similarity we explored, can improve the prediction performance of NGRHMDA, and fully demonstrated that the proposed model is feasible and effective to predict potential microbe–disease association on a large scale.

## Materials

The database explored in this work was downloaded from the Human Microbe–Disease Association Database (HMDAD, http://www.cuilab.cn/hmdad) in Sep, 2016 [33]. In the most previous studies from which the data of HMDAD database collected, microbe–disease associations were discovering from genus-level information by using 16s RNA sequencing techniques. And for those microbes which were detected in an above genus level, HMDAD keeps the original names. In total, there are 483 microbe–disease associations collected in HMDA by exploring 61 public publications. We further removed the redundant associations, and as a result, there are 450 distinct microbe–disease associations (covering 39 human diseases and 292 microbes) remained in the final dataset.

## Methods

### Neighbor-based prediction model

In the field of recommendation system, collaborative filtering (CF) was proposed to make automatic predictions about the interests of users by considering personal preferences and user and item attributes. There are two main categories of memory-based CF: one is user-based recommendation and the other is item-based recommendation [34, 35]. User-based CF is a heuristic which suggests products by searching similar users while item-based CF makes prediction by considering item similarity. Two methods have similar implementation but consider the different perspectives to make predictions.

In this work, we combined user-based and item-based CF to compute the association possibility for each microbe–disease pairs by considering other pairs sharing the same microbes/diseases, which we call “neighbors”. For measuring disease similarity, we combined Gaussian kernel-based and symptom-based similarity into an integrated one. We constructed a microbe–disease adjacent matrix based on HMDAD dataset as *A* in which *A*_*ij*_ denotes the association between disease *i* and microbe *j* (1 denotes associated and 0 denotes non-associated). Gaussian kernel-based disease similarity can be computed as follow:

1 $$ DS_{Gaussian} (i,j) = \exp \left( { - \frac{{\left\| {A_{i,*} - A_{j,*} } \right\|^{2} }}{{\gamma_{d} }}} \right) $$

where

2 $$ \gamma_{d} = {{\gamma^{\prime}_{d} } \mathord{\left/ {\vphantom {{\gamma^{\prime}_{d} } {\left( {\frac{1}{{n_{d} }}\sum\nolimits_{k = 1}^{{n_{d} }} {\left\| {A_{k,*} } \right\|^{2} } } \right)}}} \right. \kern-0pt} {\left( {\frac{1}{{n_{d} }}\sum\nolimits_{k = 1}^{{n_{d} }} {\left\| {A_{k,*} } \right\|^{2} } } \right)}} $$

Here, *γ*_*d*_ is a normalized Gaussian standard deviation based on the disease vectors and parameter input *γ*_*d*_ *′* (*γ*_*d*_ *′* was set as 0.5); *A*_k,*_ denotes the *k*-th row vector of matrix A; *n*_*d*_ is the number of diseases in HMDAD database (here, *n*_*d*_ = 39). By this way, a 39 × 39 disease similarity matrix can be constructed. In addition, we further introduced the symptom-based disease similarity scores which were previously proposed based on co-occurrence of disease/symptom terms in PubMed bibliographic records by Zhou et al. [36]. And then an integrated disease similarity matrix was constructed by averaging:

3 $$ DS = \frac{{DS_{Gaussian} + DS_{symptom} }}{2} $$

Similarly, microbe similarity matrix was constructed by computing Gaussian distances:

4 $$ MS(i,j) = \exp \left( { - \frac{{\left\| {A_{*,i} - A_{*,j} } \right\|^{2} }}{{\gamma_{m} }}} \right) $$

where

5 $$ \gamma_{m} = {{\gamma^{\prime}_{m} } \mathord{\left/ {\vphantom {{\gamma^{\prime}_{m} } {\left( {\frac{1}{{n_{m} }}\sum\nolimits_{k = 1}^{{n_{m} }} {\left\| {A_{*,k} } \right\|^{2} } } \right)}}} \right. \kern-0pt} {\left( {\frac{1}{{n_{m} }}\sum\nolimits_{k = 1}^{{n_{m} }} {\left\| {A_{*,k} } \right\|^{2} } } \right)}} $$

where *γ*_*m*_ is a normalized Gaussian standard deviation based on the microbe vectors and parameter input *γ*_*d*_ *′* (*γ*_*d*_ *′* was set as 0.5); A_*,k_ denotes the *k*-th column vector of matrix A; *n*_*m*_ is the number of microbes in HMDAD database (here, *n*_*m*_ = 292). For now, there has been no scoring method proposed for microbe functional similarity. And functional similarity could not be explained solely by homology and phylogenetic relatedness. We did not introduce additional microbial similarity as disease did. Based on the computed microbe and disease similarity matrix, we computed the association possibilities by using user-based and item-based CF. Here, microbes and diseases were regarded as “items” and “users” respectively. Given a microbe–disease pair (say *d*_*i*_ and *m*_*j*_), its association possibility was computed as follow:

6 $$ S_{disease} (d_{i} ,m_{j} ) = \frac{{\sum\nolimits_{k = 1}^{{n_{d} }} {DS(d_{i} ,d_{k} ) \cdot A_{k,j} } }}{{n_{d} }} $$

7 $$ S_{microbe} (d_{i} ,m_{j} ) = \frac{{\sum\nolimits_{k = 1}^{{n_{m} }} {MS(m_{j} ,m_{k} ) \cdot A_{i,k} } }}{{n_{m} }} $$

And the final prediction matrix (say *NS*) was computed based on the average of *S*_*disease*_ and *S*_*microbe*_:

8 $$ NS(d_{i} ,m_{j} ) = \frac{{S_{disease} (d_{i} ,m_{j} ) + S_{microbe} (d_{i} ,m_{j} )}}{2} $$

### Graph-based prediction model

Since user-item associations can be easily represented in a bipartite graph, there are an increasing number of recommended algorithms proposed based on graph-based methods [37–39]. Most of these models performed random walk algorithms like PersonalRank [40] to characterize the similarity between nodes of the user-item network, and links between users who share high rating for some items are more likely to accumulate walk counts because random walk favors large-weighted connections. However, the current version of HMDAD database is relatively small and sparse, which would lead long walks to be meaningless. Therefore, we adopted a two-step diffusion approach on the microbe–disease bipartite graph instead. In order to take microbe and disease similarities into account, we constructed two new integrated adjacency matrixes (i.e. *A*_*d*_ and *A*_*m*_) based on symptom-based disease similarity and Gaussian kernel-based microbe similarity:

9 $$ A_{d} = DS \cdot A $$

10 $$ A_{m} = A \cdot MS $$

In this way, two new adjacent matrixes with the same size of *A* could be constructed. In the first step of this diffusion approach, each disease node would be assigned weights based on the degrees of its associated microbes and the two new adjacent matrixes. In other words, microbe nodes would transfer their correlation degrees, which are recorded in A_m_ and A_d_, to their associated diseases:

11 $$ s(d_{i} ) = \alpha \sum\limits_{j = 1}^{{n_{m} }} {\frac{{A_{m(i,j)} \cdot A_{(*,j)} }}{{\sum\nolimits_{t = 1}^{{n_{d} }} {A_{m(t,j)} } }}} + (1 - \alpha )\sum\limits_{j = 1}^{{n_{m} }} {\frac{{A_{d(i,j)} \cdot A_{(*,j)} }}{{\sum\nolimits_{t = 1}^{{n_{d} }} {A_{d(t,j)} } }}} $$

Here, *s(d*_*j*_ *)* denotes to the *n*_*d*_ × 1 weight vector of disease node *d*_*j*_ assigned by its connected microbe nodes; *A*_*m(i,j)*_ and *A*_*d(i,j)*_ denote the entities in the row *i* and column *j* of *A*_*m*_ and *A*_*d*_ matrix, respectively; *α* (*α* was set as 0.5) is a damping factor to balance the contribution between *A*_*m*_ and *A*_*d*_. In the second step, the weight information of disease nodes would return back to their associated microbe nodes in a similar way with Eq. (11).

12 $$ s'(m_{j} ) = \beta \sum\limits_{k = 1}^{{n_{d} }} {\frac{{A_{m(k,j)} \cdot s(d_{k} )}}{{\sum\nolimits_{t = 1}^{{n_{m} }} {A_{m(k,t)} } }}} + (1 - \beta )\sum\limits_{k = 1}^{{n_{d} }} {\frac{{A_{d(k,j)} \cdot s(d_{k} )}}{{\sum\nolimits_{t = 1}^{{n_{m} }} {A_{d(k,t)} } }}} $$

Here, *β* (*β* was set as 0.5) is a damping factor to balance the contribution between *A*_*m*_ and *A*_*d*_. In this way, *s′(mj)* could be constructed as a *n*_*d*_ × 1 vector which records the association possibilities of *m*_*j*_ to each disease can be computed. As a result, the final prediction matrix based on this graph-based diffusion method (say GS) can be constructed by jointing *n*_*m*_ column vectors of s′ as follow:

13 $$ GS = \left[ {s^{\prime}\left( {m_{1} } \right),s^{\prime}\left( {m_{1} } \right), \ldots ,s^{\prime}\left( {m_{{n_{m - 1} }} } \right),s^{\prime}\left( {m_{{n_{m} }} } \right)} \right] $$

### Combined recommendation model for microbe–disease associations

**Fig. 1:**
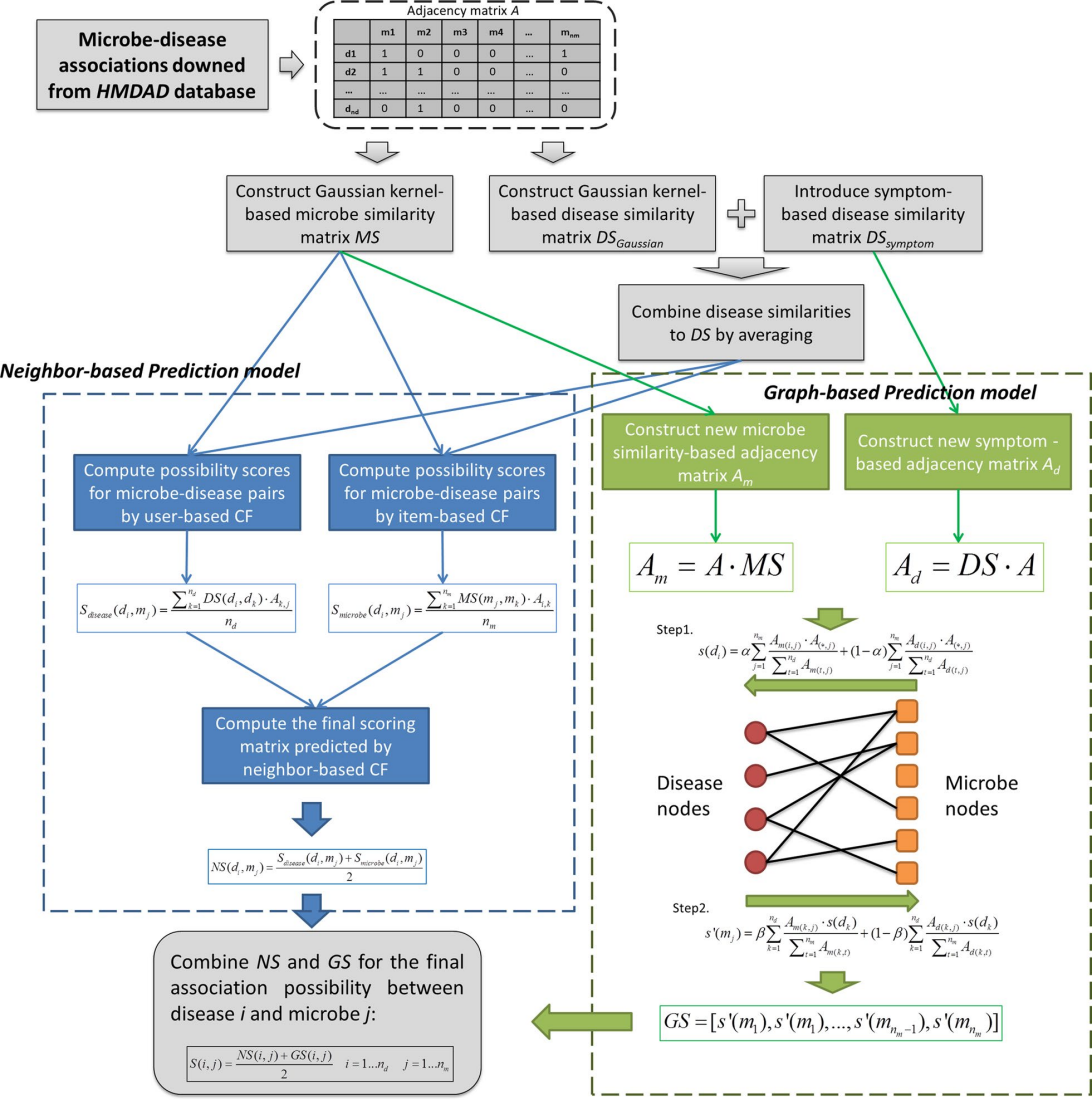
Flowchart of NGRHMDA model

Recent research in the field of recommendation system has demonstrated that the ensemble strategy can improve the performance of basic prediction model in some case [41–43]. There are a variety of recommendation algorithms have been proposed for different purposes and considerations, and the hybrid models can overcome some problems of the single model such as cold start and the sparsity problem. In this work, the two single proposed prediction models make prediction from distinct perspectives: neighbor-based CF tries to consider the similar neighbor and graph-based scoring method tries to utilize the topological information of microbe–disease bipartite graph (see Fig. 1). Therefore, it would be promising to combines them into an integrated prediction result. Given two scoring matrixes predicted by neighbor-based and graph-based model (say *NS* and *GS*), we computed the final association possibilities for each microbe–disease pairs by simply taking the average since NS and GS share the same size:

14 $$ S\left( {i,j} \right) = \frac{{NS\left( {i,j} \right) + GS\left( {i,j} \right)}}{2}\;\quad i = 1 \ldots n_{d} \quad j = 1 \ldots n_{m} $$

Here, the entity of *S(i,j)* denotes the final association possibility between disease *i* and microbe *j* predicted by the combined model.

## Results

### Leave-one-out cross validation

To evaluation the prediction ability of our NGRHMDA model, we here implemented LOOCV by using the proposed model to compute the association possibilities of microbe–disease pairs in HMDAD database. Specifically, each recorded microbe–disease association would be used as testing sample and further predicted by training the other known microbe–disease associations. And we computed the rank of each left-out testing sample by considering all uncertain microbe–disease pairs which cannot find any known relevance evidences as candidate samples. The predicted score which obtained a higher rank than the given threshold would be regarded as a successful prediction. We further computed the receiver-operating characteristics (ROC) curves of each prediction experiment based on the corresponding true positive rates (TPRs, sensitivity) and the false positive rates (FPRs, 1-specificity) with different thresholds. Here, sensitivity denotes the percentage of the testing samples obtaining higher ranks than the given threshold, and specificity means the percentage of the rest testing samples with lower ranks than the threshold. We finally computed the areas under ROC curve (AUC) to evaluate the prediction performance numerically. AUC value of 1 indicates a perfect perdition while that of 0.5 demonstrate purely random performance.

**Fig. 2:**
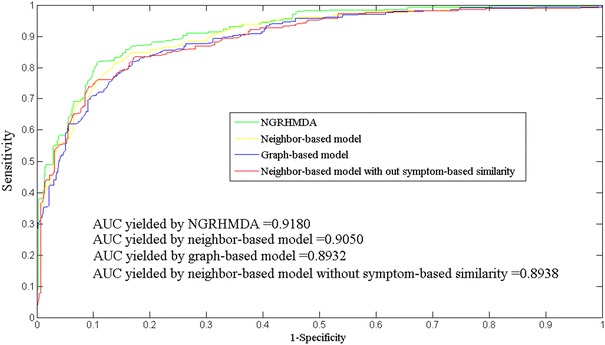
Prediction performance of NGRHMDA and three single models in terms of ROC curve and AUC based on leave-one-out cross validation

To evaluate the efficiency of the hybrid approach, we performed the microbe–disease association prediction on HMDAD database by using the two single models (i.e. neighbor-based and graph-based model) and their combined model, respectively. In addition, we further explored the effectiveness of additional information of symptom-based disease similarity by simply removing it from the neighbor-based model. As a result, the comparison results demonstrated the effectiveness of our hybrid approach as well as the introduction of other different similarity information (see Fig. 2). Specifically, NGRHMDA model obtained the best performance among these four model, yielding AUC of 0.9111 while the other two single models, neighbor-based and graph-based model, yielded AUCs of 0.9050 and 0.8932, respectively. In addition, the introduction of symptom-based disease similarity was shown to bring obvious improvement in the prediction performance in terms of the increased AUC value from the basic neighbor-based model.

### Fivefold cross validation

To further evaluate the prediction accuracy and stability, fivefold cross validation was also implemented on HMDAD database. Specifically, all the recorded micro-disease associations were randomly divided into 5 roughly equal-sized parts of which 4 were used for model learning and the rest one was used as the testing samples for model evaluation. Similar with LOOCV, all the uncertain microbe–disease pairs without known relevance evidences were regarded as potential candidates. To decrease the bias brought from the random sample division, fivefold cross validation was repeated 100 times by randomly dividing the samples in each time. As a result, NGRHMDA model yielded the highest average AUC of 0.9023 ± 0.0031 among the four models; graph-based model yielded an average AUC of 0.8871 ± 0.0026; neighbor-based model yielded an average AUC of 0.8935 ± 0.0041. Without introducing the symptom-based disease similarity, the performance of basic neighbor-based model dropped to an average AUC of 0.8834 ± 0.0034 (see Table 1).

**Table 1:** Performance comparison among four different computation models in the framework fivefold cross validation

Method	Fivefold cross validation result
NGRHMDA	0.9023 ± 0.0031
Graph-based single model	0.8871 ± 0.0026
Neighbor-based single model with symptom-based similarity	0.8935 ± 0.0041
Neighbor-based single model without symptom-based similarity	0.8834 ± 0.0034

The prediction performance has demonstrated the reliable and effective predictive ability of NGRHMDA for microbe–disease associations by only using the known microbe–disease associations and symptom-based disease similarity. And the low standard deviation of AUC yielded by NGRHMDA suggests the performance stability improved by the adopted hybrid approach. Therefore, we implemented NGRHMDA on HMDAD database to fill the microbe–disease adjacent matrix and prioritize the candidate microbes for each kind of disease. The predicted results were publicly released, which may provide valuable insights and clues for future microbial experiments and clinical research (see Additional file 1: Table S1). It is anticipated that the most potential microbe–disease pairs with high ranks would be verified by the future studies.

### Comparison with other methods

In this section, in order to evaluate the effectiveness of the proposed model, we compare the prediction performance of NGRHMDA model with some other prediction techniques including singular value decomposition (SVD), latent factor model (LFM) and Katz method. We simply performed SVD on the microbe–disease adjacency matrix and reconstructed it to fill the values of uncertain samples. Aside from neighborhood and graph-based methods, latent factor model is becoming a popular model for collaborative filtering in the field of recommendation system. It is based on a matrix factorization method and predicts ranks by optimizing users’ and items’ latent factors (also called latent features) [44, 45]. We here utilize the standard LFM method on HMDAD database by setting the size of latent factors as 100 and using gradient descent to optimize the latent factor matrixes. Katz was also explored for the performance comparison, which is a traditional and popular social network analysis method. It was also previously used for develop prediction model for microRNA-disease [46] and gene-disease associations [47]. We here combined Katz method with symptom-based disease similarity and Gaussian-kernel similarity to perform microbe–disease association prediction on HMDAD database.

**Fig. 3:**
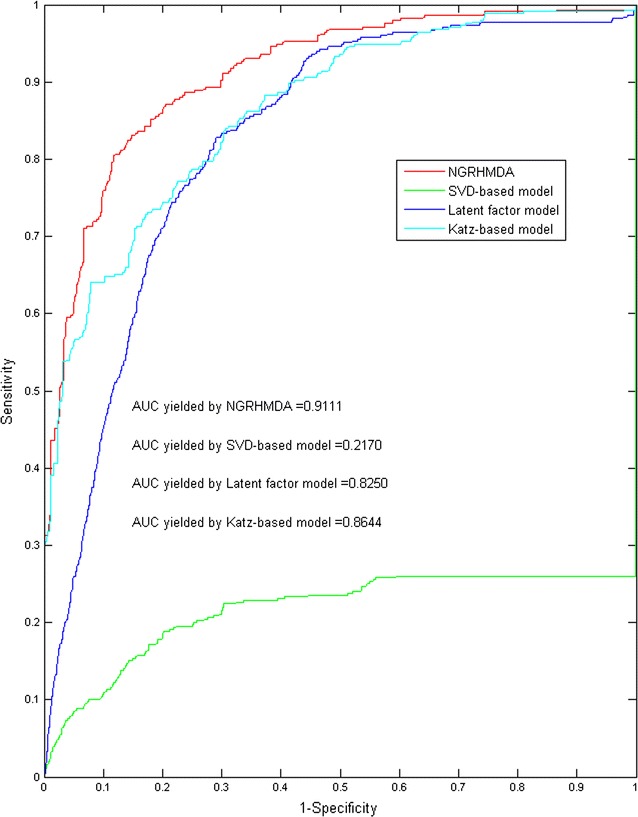
Comparison results of NGRHMDA with SVD-based, LFM-based and Katz-based prediction models in terms of ROC curve and AUC based on leave-one-out cross validation

To evaluate the prediction performance of the comparison experiment, LOOCV was implemented and the corresponding ROC curves and AUC values were computed (see Fig. 3). As a result, the proposed model, NGRHMDA, yielded the highest AUC of 0.9111; SVD-based model yielded AUC of 0.2170; latent factor model yielded AUC of 0.8250; and Katz-based model yielded 0.8644. The comparison result further demonstrated the promising prediction ability of NGRHMDA for microbe–disease associations.

### Correlation analysis of microbe and disease clusters

For the purpose of assessing the effectiveness of NGRHMDA, in this section, we further investigate into the common pattern of the microbes associated with each single type of disease and, inversely, the diseases associated with each single type of microbe. Specifically, we used the microbe–microbe similarity scores to represent a type of microbe with a feature vector. For example, with a constructed microbe–microbe similarity matrix *MS* in which entity *MS(i,j)* denotes the similarity between the *i*-th and the *j*-th microbe, the feature vector of the first type of microbe would be the first column vector of *MS* matrix. In a similar way, the feature vectors of diseases could be obtained from the *DS* matrix.

**Fig. 4:**
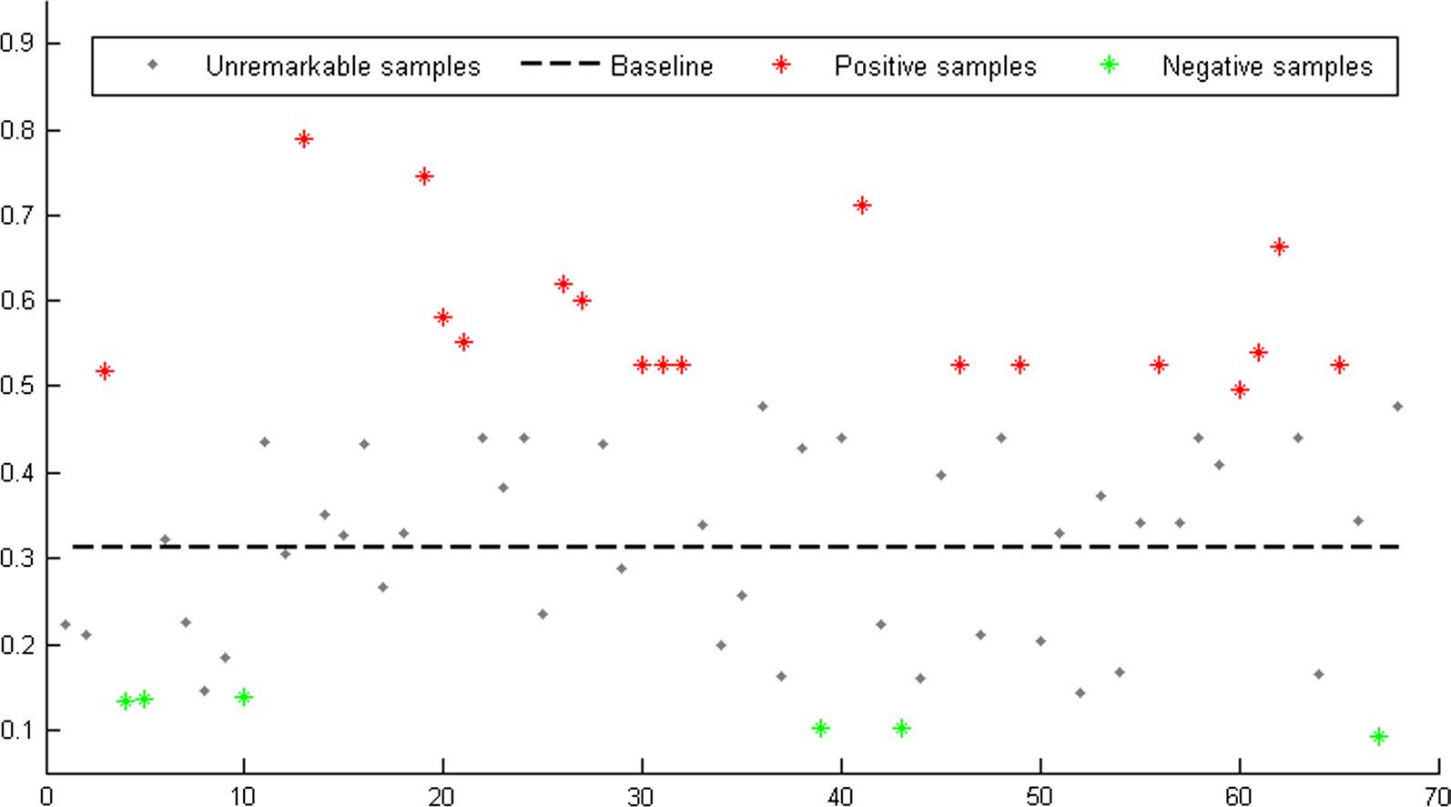
Correlation of disease clusters associated with single type of microbes

For those 68 types of microbes which have more than two records in HMDAD database, we compute the correlation scores of their associated disease cluster and take the average. The average of correlation scores measures how similar the different diseases associated with the same type microbe are. We regard the mean of autocorrelation matrix of *DS* matrix as the baseline. In addition, in order to draw a more reliable conclusion, we highlight the samples having significantly higher or lower correlation score than the baseline by using a difference threshold of a standard deviation (see the red and green star points in Fig. 4). As a result, we found that 78.2% (18/23) highlighted samples were found to be consistent with our assumption that microbes tend to get involved in similar diseases. In addition, our assumption is also supported by the result that the average correlation score of associated disease clusters for single type of microbe achieves 0.3690, which is significantly higher than the baseline of 0.3121.

**Fig. 5:**
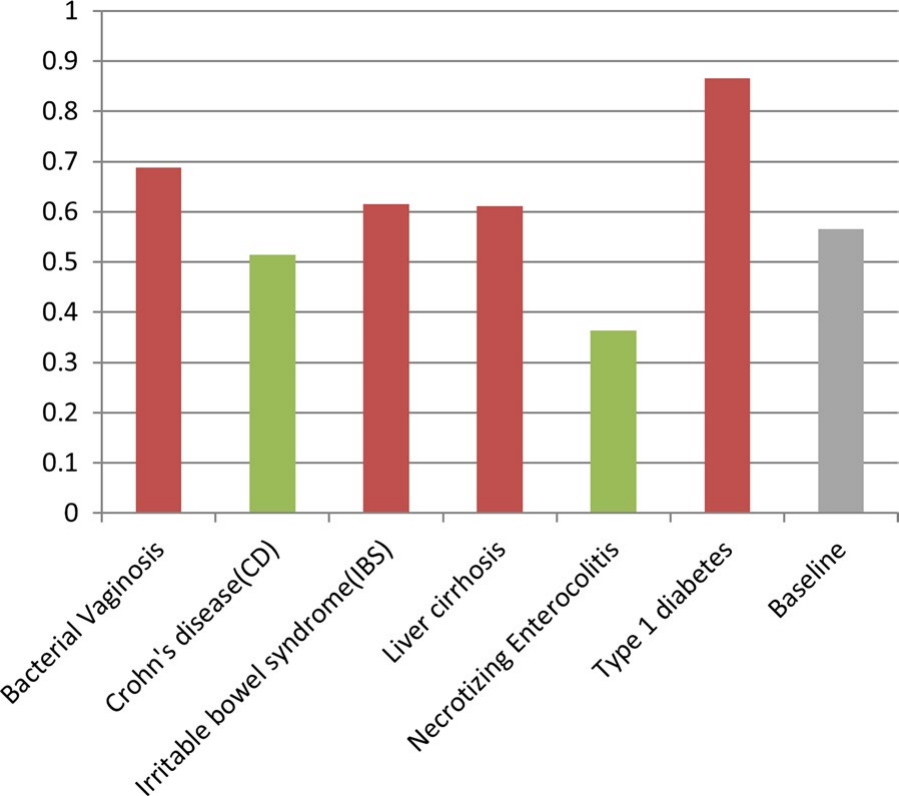
Correlation of microbe clusters associated with single type of diseases

Besides, a similar statistics analysis was also implemented on the microbe clusters associated with each single type disease (see Fig. 5). Considering the diseases recorded in HMDAD database have associations with approximately 11 types of microbes in average, we thus focus only on the 6 types of diseases which have more than 10 records. As a result, 66.7% (4/6) samples were found to be consistent with our assumption that diseases tend to be associated with similar microbes. In addition, the average correlation score of associated microbe clusters for single type of disease achieves 0.6098, which is significantly higher than the baseline of 0.5661. It should be noted that the adjacency matrix for known microbe–disease associations is still far from complete due to the current limited knowledge. Therefore, it is anticipated that the conclusion could be confirmed more reliably with more clinical observations in the future.

## Disccusion

There are an increasing number of clinical evidences showing that the involvement of specific microbe with abnormal levels can significantly influence the development of various kinds of human diseases including the noninfectious ones. Detecting the disease-causing microbes for specific diseases can boot the understanding of disease mechanism and provide valuable information for the prevention, diagnosis and treatment of various diseases. However, little effort has been devoted to develop computational model for predicting microbe–disease associations on a large scale. In this work, we explored the HMDAD database which collects detected microbe–disease associations from previously published experimental reports to predict the most potential microbial candidates for different diseases. NGRHMDA was proposed by combining two single recommendation prediction models which are based on neighbor information and graph topology, respectively. In addition, it has an open frame which allows different types of microbe/disease similarity (e.g. symptom-based disease similarity, disease phenotypic similarity and disease semantic similarity) to be introduced by combining with the Gaussian-kernel based similarity. Leave-one-out and fivefold cross validation were implemented for performance evaluation. As a result, NGRHMDA yielded reliable results with AUCs of 0.9111 and 0.9023 ± 0.0031 in the evaluation frameworks of LOOCV and fivefold CV, respectively, which fully demonstrated the effectiveness of the proposed model. We anticipate that the microbe–disease associations which were predicted as potential candidates with high ranks will be confirmed by future experimentally observations.

NGRHMDA solves the problem of predicting disease-causing microbes in a similar way with recommendation system which predicts ratings for items that the user may have an interest in. That is, NGRHMDA predicted association possibilities for each microbe–disease pair by regarding microbes and diseases as “items” and “users” respectively based on the assumption that functionally similar microbes tend to be involved in the mechanism of similar diseases. Two single prediction models combined by NGRHMDA make prediction from different perspectives, and therefore are expected to provide comprehensive information based on the training data. By using the hybrid approach, NGRHMDA was demonstrated to have obvious performance improvement from the single models in terms of prediction accuracy and stability. In addition, the introduction of additional disease similarity also proves to be useful for the performance improvement. Compared with other prediction techniques, NGRHMDA model has obvious advantages with high prediction performance for microbe–disease association prediction. As an unsupervised learning model, NGRHMDA does not need any negative samples for learning and can be implemented to microbes/diseases with the information of known associated diseases/microbes. Therefore, it is anticipated that NGRHMDA can be used as a feasible and effective computational tool for searching microbial candidates for various disease on a large scale.

However, some limitations still exist in the current version of NGRHMDA. First, it still needs manual intervention to adjust model parameters such as the two damping factors (i.e. *α* and *β*) of graph-based model, which may hinder the prediction performance when performing on different databases. In addition, similar with the diversity problem of recommendation models, NGRHMDA may excessively “recommend” some well-studied microbes, which are known to be associated with many diseases, to the query disease. Finally, NGRHMDA cannot be applied to new diseases/microbes which have no any known microbe/disease association. Further name matching method for disease and microbe inputs may solve this problem to some extent.

## Additional file

**Additional file 1: Table S1.** We publicly released the predicted of microbes for each disease, which may offer valuable information and clues for biological experiments.

## Abbreviations

COPD: chronic obstructive pulmonary disease
MS: mass spectrometry
CDI: clostridium difficile infection
NGRHMDA: neighbor- and graph-based combined recommendation model for human microbe–disease association prediction
LOOCV: lease-one-out cross validation
Fivefold CV: fivefold cross validation
HMDAD: Human Microbe–Disease Association Database
CF: collaborative filtering
ROC: receiver-operating characteristic
TPR: true positive rate
FPR: false positive rate
AUC: the areas under ROC curve
SVD: singular value decomposition
LFM: latent factor model
